# Roles of Sialic Acid, AXL, and MER Receptor Tyrosine Kinases in Mumps Virus Infection of Mouse Sertoli and Leydig Cells

**DOI:** 10.3389/fmicb.2020.01292

**Published:** 2020-06-29

**Authors:** Fei Wang, Ran Chen, Qian Jiang, Han Wu, Maolei Gong, Weihua Liu, Xiaoqin Yu, Wenjing Zhang, Ruiqin Han, Aijie Liu, Yongmei Chen, Daishu Han

**Affiliations:** Peking Union Medical College, School of Basic Medicine, Chinese Academy of Medical Sciences, Institute of Basic Medical Sciences, Beijing, China

**Keywords:** mumps virus, testis, sialic acid, AXL, MER

## Abstract

The mumps virus (MuV) causes epidemic parotitis. MuV also frequently infects the testis and induces orchitis, an important etiological factor contributing to male infertility. However, mechanisms underlying MuV infection of the testis remain unknown. Here, we describe that sialic acid, AXL, and MER receptor tyrosine kinases regulate MuV entry and replication in mouse major testicular cells, including Sertoli and Leydig cells. Sialic acid, AXL, and MER were present in Sertoli and Leydig cells. Sialic acid specifically mediated MuV entry into Sertoli and Leydig cells, whereas both AXL and MER facilitated MuV replication within cells through the inhibition of cellular innate antiviral responses. Mechanistically, the inhibition of type 1 interferon signaling by AXL and MER is essential for MuV replication in Sertoli and Leydig cells. Our findings provide novel insights into the mechanisms behind MuV infection and replication in the testis.

## Introduction

The mumps virus (MuV) is a pathogen that causes epidemic mumps, a painful parotitis (Latner and Hickman, 2015). MuV also has tropism for the testis, thereby frequently inducing orchitis, which may result in male infertility (Litman and Baum, 2010). Although a large vaccination program has remarkably reduced the spread of the pathogen and declined the incidence of mumps, a global outbreak of mumps has been recently reported in the vaccinated population (Vandermeulen et al., 2004; Castilla et al., 2007; Cortese et al., 2008; Dayan et al., 2008; Marin et al., 2008; Bangor-Jones et al., 2009; Centers for Disease Control and Prevention, 2009; Patel et al., 2017; Willocks et al., 2017). Knowledge gaps in the basic biology of MuV hinder progress in eliminating mumps (Ramanathan et al., 2018). The mechanisms underlying MuV infection and replication in target cells need clarification, which may aid in the development of preventive and therapeutic strategies for MuV-induced pathogenesis.

MuV is an enveloped and non-segmented negative-sense RNA virus (Rubin et al., 2015). The MuV genomic RNA is encapsidated by a nucleoprotein (NP). MuV infection in children usually results in mumps, an acute inflammation of the parotid glands (Anderson et al., 1987). In addition to mumps, MuV infection may result in inflammation in multiple other organs, including the central nervous system, pancreas, kidney, ovary, and testis (Rubin and Sauder, 2013). Orchitis is the most common complication of mumps, occurring in about 20–30% of mumps cases (Masarani et al., 2006). While mumps orchitis in childhood is self-limiting and rarely leads to sterility, MuV infection in post-pubertal men frequently results in chronic orchitis, one important etiological factor of male infertility. The recovery of MuV from the testis of mumps orchitis patients confirms that MuV directly infects the testis (Bjorvatn, 1973). MuV can infect various testicular cells (Wu et al., 2017). MuV triggers innate immune responses in mouse Sertoli and Leydig cells, thereby inducing the expression of pro-inflammatory cytokines and impairing testosterone synthesis (Wu et al., 2016). In Sertoli cells, MuV-induced pro-inflammatory cytokines, such as TNFA and CXCL10, induce male germ cell apoptosis (Jiang et al., 2017). MuV infection of Sertoli cells impairs the integrity of the blood-testis barrier through the induction of TNFA (Wu et al., 2019). While MuV infects testicular cells and impairs testicular functions, the mechanisms underlying MuV infection and replication in the testicular cells remain to be elucidated.

AXL and MER belong to a subfamily of receptor tyrosine kinases. AXL and MER are involved in the maintenance of immune homeostasis and play important roles in regulating the testicular immune privilege (Rothlin et al., 2015; Deng et al., 2016). Several studies have demonstrated that AXL facilitates infection of a broad spectrum of viruses, including Ebola (Shimojima et al., 2006), vaccinia (Morizono et al., 2011), dengue (Meertens et al., 2012), influenza (Shibata et al., 2014), and Zika (Richard et al., 2017) viruses (ZIKV). Both AXL and MER are abundantly expressed in Sertoli and Leydig cells and inhibit innate antiviral responses (Wang et al., 2005; Sun et al., 2010; Shang et al., 2011). The potential roles of AXL and MER in regulating MuV infection in Sertoli and Leydig cells have yet to be investigated. Sialic acid facilitates multiple viral infections that are of significant public concern, including avian influenza virus (Weis et al., 1988), picornavirus (Liu et al., 2015), and coronavirus (Li et al., 2017). Recent studies have demonstrated that sialic acid on the cell surface mediates MuV and ZIKV infection (Kubota et al., 2016; Tan et al., 2019). Whether sialic acid plays a role in regulating MuV infection in testicular cells is unknown. In the present study, we elucidate the roles of sialic acid, AXL, and MER in MuV infection and replication in mouse Sertoli and Leydig cells.

## Materials and Methods

### Animals

C57BL/6J mice were obtained from the Laboratory Animal Center of Peking Union Medical College (Beijing, China). *Axl* or *Mer* single knockout (*Axl* ^−/−^ and *Mer* ^−/−^) mice were kindly provided by Dr. Qingxian Lu (University of Louisville, Louisville, KY, USA). *Axl* and *Mer* double knockout (*Axl* ^−/−^ *Mer* ^−/−^) mice were obtained by mating *Axl* ^−/−^ and *Mer* ^−/−^ mice, and wild-type (WT) mice were generated by backcrossing *Axl* ^−/−^ *Mer* ^−/−^ mice to C57BL/6J mice. *Ifna/b* receptor knockout (*Ifnar1* ^−/−^) mice were purchased from the Jackson Laboratory (Bar Harbor, ME, USA). All of the mice were maintained in a specific pathogen-free facility with a 12/12 h light/dark cycle, and they were provided with food and water *ad libitum*. Mice were handled according to the Guideline for the Care and Use of Laboratory Animals established by the Chinese Council on Animal Care [permit number: SCXK (Jing) 2007-0001]. The experimental procedures were approved by the Institutional Animal Care and Use Committee of the Institute of Basic Medical Sciences, Chinese Academy of Medical Sciences (Beijing, China).

### Antibodies and Major Reagents

Polyclonal rabbit anti-MX1 (sc-50509), monoclonal mouse anti-MuV-NP (sc-57922), and anti-OAS1 (sc-365072) antibodies were purchased from Santa Cruz Biotechnology (Dallas, TX, USA). Polyclonal rabbit anti-ISG15 antibody (#2743) was purchased from Cell Signaling Technology (Beverly, MA, USA). Polyclonal rabbit anti-3β-HSD (OAGA02009) was purchased from Aviva System Biology (San Diego, CA, USA). Monoclonal rat anti-MER (14-5751-82) antibody and Polyclonal rabbit anti-Wilms tumor nuclear protein 1 (WT1) antibody (PA5-16879) were purchased from Thermo Fisher Scientific (Waltham, MA, USA). Monoclonal mouse anti-β-Actin antibody (66009-1-lg) and anti-α-tubulin (F2168) were purchased from Sigma-Aldrich (St. Louis, MO, USA). Polyclonal rabbit anti-AXL antibody (ab227871) was purchased from Abcam (Cambridge, UK). 4',6'-diamidino-2-phenylindole (DAPI), tetramethyl rhodamine isothiocyanate (TRITC)-, and fluorescein isothiocyanate (FITC)-conjugated secondary antibodies were purchased from Zhongshan Biotechnology (Beijing, China). α2,3-Sialidase (4455) was purchased from TaKaRa Bio Inc. (Kyoto, Japan). Biotinylated *Maackia amurensis* Lectin II (MAL II) (B-1265) was purchased from Vector Laboratories (Burlingame, CA, USA). LDC1267 (S7638) was purchased from Selleckchem (Houston, TX, USA).

### MuV Preparation

MuV (strain SP-A) was isolated from mumps patients (Liang et al., 2014), and provided by Prof. Qihan Li (The Institute of Medical Biology, Chinese Academy of Medical Science, Kunming, China). MuV was amplified and titrated in Vero cells. Briefly, Vero cells (5 × 10^6^) were seeded in 100 mm culture dishes with 10 ml Dulbecco’s modified Eagle medium (DMEM; Thermo Fisher Scientific, Waltham, MA, USA) supplemented with 10% fetal calf serum (FCS; Thermo Fisher Scientific). After 24 h, the cells were infected with MuV at a multiplicity of infection (MOI) of 1.0. Seven days after MuV infection, the culture media were collected, and the cells were lysed by freezing in liquid nitrogen and thawing at 37°C three times. After centrifugation at 2,000 rpm for 10 min, the supernatants were collected. MuV preparations were retained in PBS at a density of 1 × 10^9^ plaque-forming unit (PFU)/ml and stored at −80°C until further use.

### Plaque Assay

Vero cells were seeded in 6-well plates at 2 × 10^5^ cells/well. After 24 h, the cells were infected with a serial dilution of MuV for 1 h at 37°C. The medium was removed and replaced with DMEM containing 2% FCS and 1.5% methylcellulose (Sigma-Aldrich). The cells were cultured in a humidified incubator containing 5% CO_2_ at 37°C for 7 days. The cells were stained with 1% crystal violet solution (Sigma-Aldrich) according to the manufacturer’s instructions. Plaques were counted and PFU was calculated.

### MuV Binding, Internalization, and Replication

MuV binding to Sertoli and Leydig cells was determined after incubating the cells with 50 MOI MuV on ice for 1 h. After triple washes with PBS, cells were collected for virus detection. For MuV internalization, cells were incubated with 50 MOI MuV for 1 h at 37°C and the surface-bound MuV was removed by treating cells with 0.25% trypsin (Thermo Fisher Scientific) for 5 min. The cells were collected for MuV detection. For MuV replication, cells were infected with 1.0 MOI MuV. Two hours later, the cells were washed twice with PBS, and then were cultured in fresh medium at 32°C. The cells were harvested for MuV detection each 24 h.

### Cell Isolation

Sertoli and Leydig cells were isolated from 3-week-old mice (*n* = 3 each isolation) following previously described procedures (Zhu et al., 2013). Briefly, the testes were decapsulated and incubated with 0.5 mg/ml collagenase type I (Sigma-Aldrich) in PBS at 37°C for 15 min. The interstitial cells were separated from the seminiferous tubules after filtration with 80 μm copper meshes. The interstitial cells were cultured at 32°C in F12/DMEM (Thermo Fisher Scientific) supplemented with 100 U/ml penicillin, 100 mg/ml streptomycin, and 10% FCS. Twenty-four hours later, Leydig cells were detached by treatment with 0.25% trypsin for 5 min. The purity of Leydig cells was more than 95% based on immunostaining for 3β-HSD, a marker of Leydig cells (Klinefelter et al., 1987).

The seminiferous tubules were re-suspended in collagenase type I at room temperature for 15 min to remove peritubular myoid cells. The seminiferous tubules were cut into small pieces of approximately 1 mm and incubated with 1 mg/ml hyaluronidase (Sigma-Aldrich) at 37°C for 15 min with gentle pipetting to dissociate germ cells from Sertoli cells. The cell suspensions were cultured at 32°C for 24 h and then treated with a hypotonic solution (20 mM Tris, pH 7.4) for 3 min to remove germ cells adhering to Sertoli cells. The purity of Sertoli cells was >95% based on the immunostaining for WT1, a marker of Sertoli cells (Sharpe et al., 2003).

### Immunofluorescence Staining

Cells were cultured on 35 mm culture dishes and fixed with pre-cold 4% methanol at −20°C for 3 min. After washing twice with PBS, the cells were permeabilized with 0.2% Triton X-100 (Zhongshan Biotechnology Co.) in PBS for 10 min and blocked with 5% normal goat serum (Zhongshan Biotechnology Co.) for 1 h at room temperature. The cells were subsequently incubated with primary antibodies at 4°C for 24 h. After washing twice with PBS, the cells were incubated with appropriate TRITC‐ or FITC-conjugated secondary antibodies for 1 h at room temperature. Nuclei were counterstained with DAPI according to the manufacturer’s instructions. The cells were mounted with antifade mounting medium (Zhongshan Biotechnology Co.) and observed under a confocal laser scanning microscope FV1000 (Olympus, Tokyo, Japan) or fluorescence microscope BX-51 (Olympus).

### Real-Time Quantitative RT-PCR

Total RNA was extracted from cells using Trizol reagents (Thermo Fisher Scientific) according to the manufacturer’s instructions. RNA was treated with RNase-free DNase I (Thermo Fisher Scientific) to remove genomic DNA. Total RNA (1 μg) was reverse transcribed into cDNA in 20 μl reaction mixture containing random hexamers at 2.5 μM, 2 μM of deoxynucleotide triphosphates, and 200 units of Moloney murine leukemia virus reverse transcriptase (Promega, Madison, WI, USA). The PCR was performed in 20 μl reaction mixture containing 0.2 μl of cDNA, forward and reverse primers at 0.5 μM, and 10 μl of Power SYBR Green PCR Master Mix (Applied Biosystems, Foster City, CA, USA) on an ABI PRISM 7300 real-time cycler (Applied Biosystems). Relative mRNA levels were determined using the 2^−ΔΔCt^ method as described in the Applied Biosystems User Bulletin No. 2 (P/N 4303859). The primer sequences for PCR are listed in Table 1.

**Table 1:** Primers used for real-time qRT-PCRs.

Primer pairs (5' → 3')		
Target genes	Forward	Reverse
MuV-NP	TCAGATCAATCGCATCGGGG	CTTGCGACTGTGCGTTTTGA
*Isg15*	CCAGTCTCTGACTGTGAGAGC	GCATCACTGTGCTGCTGGGAC
*Oas1*	ATTACCTCCTTCCCGACACC	CAAACTCCACCTCCTGATGC
*Mx1*	GACCATAGGGGTCTTGACCAA	AGACTTGCTCTTTCTGAAAAGCC
*Ifna*	TTCCTCAGACTCATAACCTCAGGA	ATTTGTACCAGGAGTGTCAAGGC
*Ifnb*	GACGTGGGAGATGTCCTCAAC	GGTACCTTTGCACCCTCCAGTA
*Actb*	GAAATCGTGCGTGACATCAAAG	TGTAGTTTCATGGATGCCACAG

### Western Blot

The cells were lysed in a lysis buffer containing a protease inhibitor cocktail (Sigma-Aldrich). The protein concentrations were determined using a bicinchoninic acid protein assay kit (Pierce Biotechnology, Rockford, IL, USA). Proteins (20 μg/well) were separated by 10% SDS-PAGE and then electrotransferred onto polyvinylidene fluoride membranes (Millipore, Bedford, MA, USA). The membranes were blocked with Tris-buffered saline (pH 7.4) containing 5% non-fat milk for 1 h at room temperature and then incubated with the primary antibodies for 24 h at 4°C. After washing twice with Tris-buffered saline containing 0.1% Tween-20, the membranes were incubated with HRP-conjugated secondary antibodies for 1 h at room temperature. Protein bands were visualized with an enhanced Chemiluminescence Detection Kit (Zhongshan Biotechnology Co.). β-Actin was used as the loading control.

### TCID_50_ Assay

Vero cells were cultured in 96-well plates (6 × 10^3^/well) in 100 μl of culture medium. A serial dilution of MuV was added. After 1 week, TCID_50_ values were calculated according to the Reed-Muench method.

### Cell Viability Assay

Cell viability was determined after MuV infection. Briefly, cells were cultured in 96-well plates and infected with MuV. At 2, 24, 48, and 72 h after infection, cell viability was assessed using a Cell Counting Kit 8 (CCK-8; Dalian Meilun Biotechnology Co., Ltd., Dalian, China) assay according to the manufacturer’s instructions.

### Fluorescence Staining for Sialic Acid

The cells were fixed with 4% paraformaldehyde for 5 min. After washing twice with PBS, the cells were permeabilized with 0.2% Triton X-100 in PBS for 10 min. Biotinylated *M. amurensis* lectin II (20 μg/ml) was then added to the cells for 1 h at room temperature. FITC-conjugated streptavidin (Solarbio, Beijing, China) was added to the cells at a dilution of 1:200 for 1 h at room temperature. The nuclei were stained with DAPI. The cells were mounted with antifade mounting medium and observed under a BX-51 microscope.

### Enzyme-Linked Immunosorbent Assay

The cell media were collected at 48 h after MuV infection. The cytokine levels in the media were measured using ELISA kits in accordance with the manufacturer’s instructions. ELISA kits for mouse IFNA (BMS6027) and IFNB (42400) were purchased from eBioscience (San Diego, CA, USA) and R&D Systems (Minneapolis, MN, USA), respectively.

### MuV Infection *in vivo*

For MuV infection of the testis *in vivo*, 8-week-old mice were anesthetized with pentobarbital sodium at a dose of 50 mg/kg. The testes were surgically exposed and each testis was injected with 10 μl PBS containing 1 × 10^7^ PFU of MuV using 30-gauge needle. Three mice in each group were injected.

### Statistical Analysis

Statistical difference between two comparisons was determined using Student’s *t*-test. One-way ANOVA with Bonferroni’s (selected pairs) *post hoc* test was used for multiple comparisons. The calculations were performed with SPSS version 13.0 (SPSS Inc., Chicago, IL, USA). Data were presented as the mean ± SEM of at least three experiments. A *p* < 0.05 was considered to be statistically significant.

## Results

### MuV Binding and Internalization

**Figure 1:**
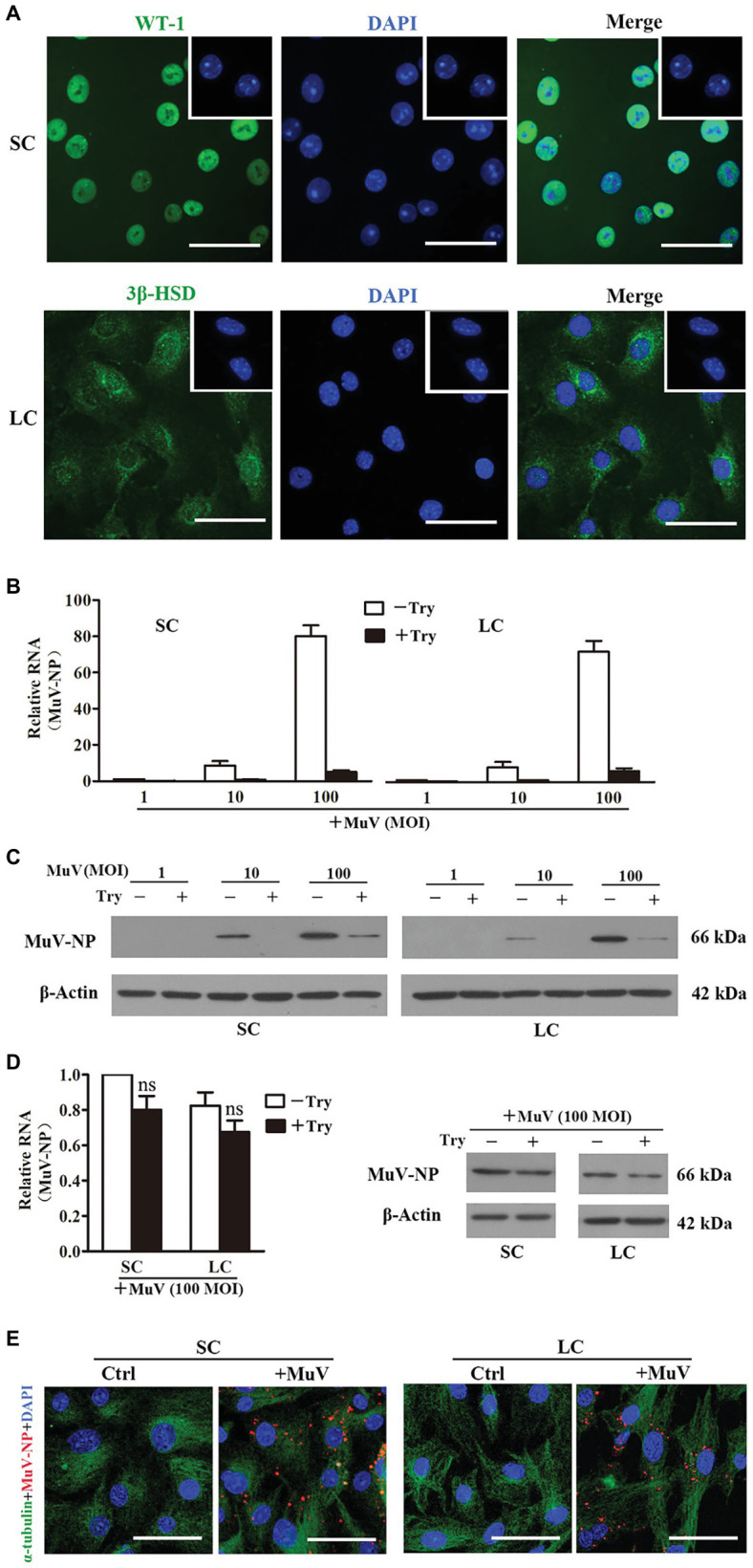
Mumps virus (MuV) binding and internalization. **(A)** Cell purity. Sertoli cells (SC) and Leydig cells (LC) were isolated from three-week-old mice. Cell purity was assessed using immunofluorescence (IF) staining of Wilms tumor nuclear protein 1 (WT-1) for SC (upper panel) and 3β-HSD (lower panel) for LC. The cellular nuclei were counterstained with 4',6'-diamidino-2-phenylindole (DAPI). Insets in the upper right corners show the negative controls, in which pre-immune rabbit sera served as FIGURE 1the primary antibodies. **(B,C)** MuV binding. SC and LC were incubated with the indicated doses (MOI) of MuV on ice for 1 h. After washing twice with PBS, cells were treated with 0.25% trypsin (Try) for 5 min. Total RNA and protein were extracted. MuV nuclear protein (MuV-NP) RNA **(B)** and protein **(C)** levels were determined by real-time qRT-PCR and Western blot, respectively. **(D,E)** MuV internalization. SC and LC were incubated with 100 MOI of MuV at 37°C for 1 h: **(D)** Cells were treated with trypsin for 5 min. MuV-NP RNA (left panel) and protein (right panel) levels were determined by real-time qRT-PCR and Western blot, respectively. β-Actin was used as internal control for qRT-PCR and loading control for Western blot. **(E)** Intracellular MuV-NP were determined by IF staining with antibodies against MuV-NP (red). Cellular plasma and nuclei were visualized using IF for α-tubulin (green) and DAPI (blue) counterstaining, respectively. Cells, incubated with MuV-free PBS served as the controls (Ctrl). Images represent at least three independent experiments. Data are presented as the mean ± SEM of three experiments. Scale bars, 50 μm. ns, not significant.

To assess the MuV infection of testicular cells, viral binding and internalization to Sertoli and Leydig cells were examined. Sertoli (Figure 1A, upper panels) and Leydig (lower panels) cells were identified by immunofluorescence staining for WT-1 and 3β-HSD, and the purity of each cell type was above 95%. Relative MuV nuclear protein (MuV-NP) RNA levels were detected by real-time qRT-PCR in Sertoli and Leydig cells 1 h after incubation with different doses of MuV on ice for MuV binding (Figure 1B). The treatment of cells with trypsin after MuV binding dramatically decreased the MuV-NP RNA levels. Western blot clearly detected the MuV-NP protein in Sertoli (Figure 1C, left panels) and Leydig (right panels) cells 1 h after MuV binding at doses of 10 and 100 MOI. The trypsin treatment remarkably reduced MuV-NP protein levels. The results suggest that MuV can efficiently bind to Sertoli and Leydig cells, and that the bound MuV can be removed by trypsin treatment. MuV internalization into Sertoli and Leydig cells was examined by measuring MuV-NP RNA (Figure 1D, left panel) and protein levels (right panel) after incubation with 100 MOI MuV for 1 h at 37°C. The trypsin treatment did not significantly reduce MuV-NP RNA and protein levels after internalization. The presence of MuV in cells after internalization was further confirmed by immunofluorescence staining for MuV-NP (Figure 1E). These observations indicate that MuV can efficiently internalize into Sertoli and Leydig cells.

### MuV Replication and Secretion

**Figure 2:**
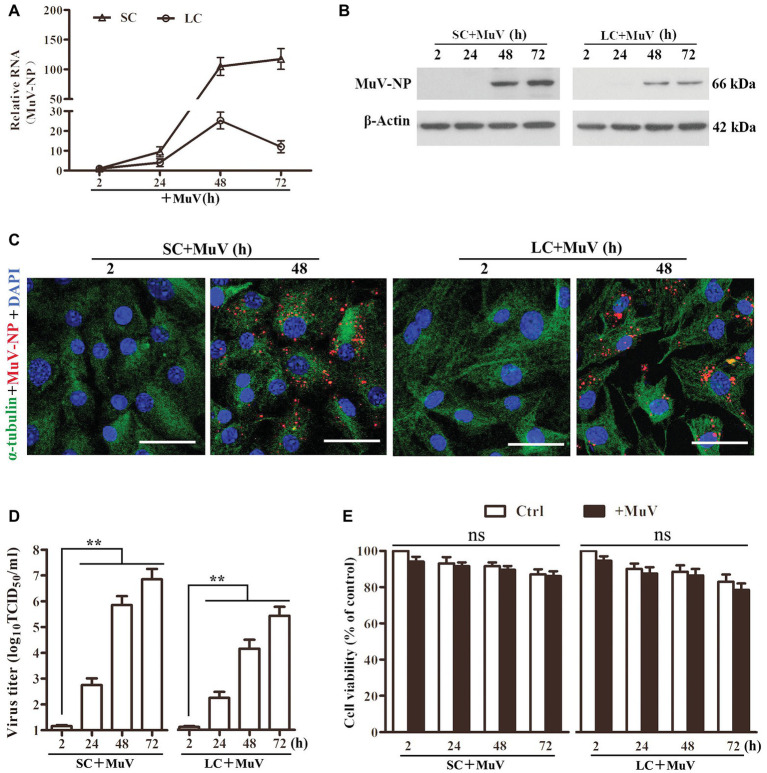
MuV replication. **(A)** MuV-NP RNA level. SC and LC were infected with 1.0 MOI of MuV for the specified hours (h). Relative MuV-NP RNA levels were examined using real-time qRT-PCR. **(B)** Protein levels of MuV-NP. SC (left panel) and LC (right panel) were infected with MuV as described in (A). Time-dependent MuV-NP protein levels were determined using Western blot. **(C)** Intracellular MuV. SC and LC were infected with 1.0 MOI of MuV for the indicated durations. Intracellular MuV were determined using IF staining for MuV-NP (red). Cellular plasma and nuclei were visualized using IF for α-tubulin (green) and DAPI (blue) counterstaining. **(D)** MuV secretion. SC and LC were infected with MuV as described in **(A)**. MuV loads in culture media were analyzed by TCID_50_ assay. **(E)** Cell viability. SC and LC were infected with MuV as described in **(A)**. Cell viability was assessed by CCK-8 assay. Images represent three independent experiments. Data are presented as the mean ± SEM of three experiments. ns, not significant, ^**^ *p* < 0.01.

To examine MuV replication in Sertoli and Leydig cells, the cells were infected with 1.0 MOI MuV. Real-time qRT-PCR results showed that the MuV-NP RNA level dramatically increased in Sertoli and Leydig cells in a time-dependent manner (Figure 2A). Western blot results demonstrated that MuV-NP protein levels were remarkably increased in Sertoli and Leydig cells 48 and 72 h after infection (Figure 2B). Both MuV-NP RNA and protein levels were higher in Sertoli cells than Leydig cells, suggesting that MuV replicated at relatively high efficiency in Sertoli cells compared to Leydig cells. Immunofluorescence staining confirmed that MuV-NP was increased in Sertoli (Figure 2C, left panels) and Leydig (right panels) cells 48 h after infection. TCID_50_ assay showed that MuV loads in the culture media of Sertoli and Leydig cells were significantly increased in a time-dependent manner (Figure 2D), suggesting that MuV particles can be secreted from Sertoli and Leydig cells. The CCK-8 assay indicated that MuV infection insignificantly impaired cell viability (Figure 2E).

### Role of Sialic Acid in MuV Internalization

**Figure 3:**
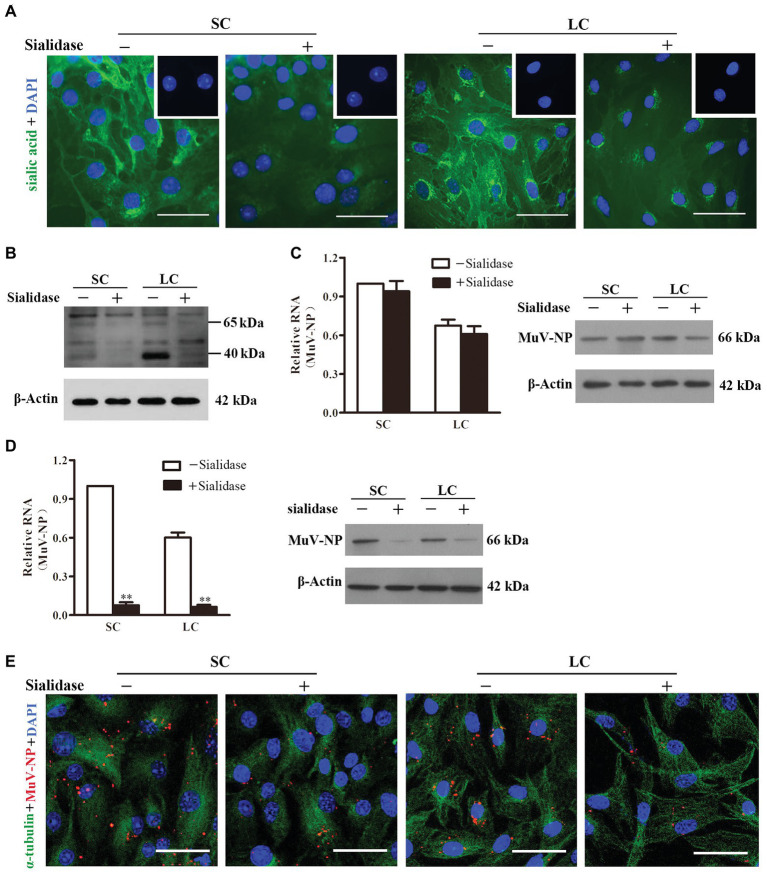
Role of sialic acid in MuV internalization. **(A)** Sialic acid on the cellular surface. SC and LC were cultured in the absence (−) and presence (+) of 0.2 U/ml α2,3-Sialidase for 24 h. Cells were stained for sialic acid using biotinylated *Maackia amurensis* lectin II, and cellular nuclei were counterstained with DAPI. Insets in the upper right corners show the negative controls, in which the biotinylated *M. amurensis* lectin II was replaced by PBS. **(B)** SC and LC were treated as described in **(A)**, and the sialic acid was detected using Western blot. **(C)** MuV binding. SC and LC were cultured in the absence and presence of sialidase for 24 h, and subsequently incubated with 100 MOI of MuV on ice for 1 h. MuV-NP RNA (left panel) and protein (right panel) levels were determined by real-time qRT-PCR and Western blot, respectively. **(D)** MuV internalization. SC and LC were treated as described in **(C)**, and incubated with 100 MOI of MuV at 37°C for 1 h. MuV-NP RNA (left panel) and protein (right panel) levels were determined. **(E)** SC and LC were treated as described as in **(D)**. Intracellular MuV were determined by IF staining for MuV-NP (red). Cellular plasma and nuclei were visualized using IF for α-tubulin (green) and DAPI (blue) counterstaining, respectively. Images represent at least three independent experiments, scale bars, 50 μm. Data are presented as the mean ± SEM of three experiments. ^**^ *p* < 0.01.

Given that trisaccharide with α2,3-linked sialic acid is a MuV receptor (Kubota et al., 2016), we examined the role of sialic acid in MuV infection of Sertoli and Leydig cells. Fluorescence staining indicated the presence of sialic acid on the surface of Sertoli (Figure 3A, left panels) and Leydig (right panels) cells. The sialic acid was efficiently removed by the treatment of cells with 0.2 U/ml α2,3-Sialidase. The presence of the sialic acid and its removal by α2,3-Sialidase were further confirmed by Western blot (Figure 3B). Real-time qRT-PCR results showed that the pre-treatment of cells with α2,3-Sialidase did not alter the RNA level of MuV-NP binding to Sertoli and Leydig cells (Figure 3C, left panel). Western blot results confirmed that the protein levels of MuV-NP binding to Sertoli and Leydig cells were comparable in the absence and the presence of α2,3-Sialidase (Figure 3C, right panel). However, the internalized MuV-NP RNA (Figure 3D, left panel) and protein (right panel) levels in both Sertoli and Leydig cells were significantly decreased by the presence of α2,3-Sialidase. The decreased MuV internalization by α2,3-Sialidase was confirmed using immunofluorescence staining for MuV-NP (Figure 3E). These results suggest that sialic acid facilitates MuV internalization into Sertoli and Leydig cells, but does not affect MuV binding to these cells.

### Role of AXL and MER in MuV Replication

**Figure 4:**
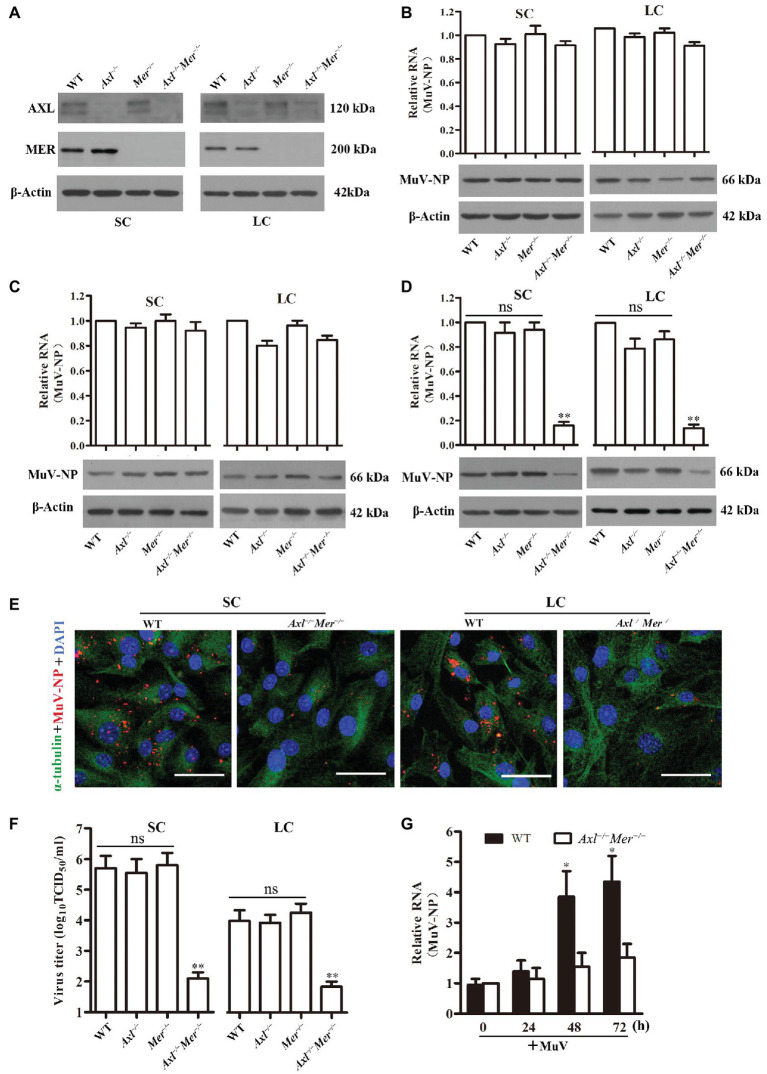
Role of AXL and MER in MuV replication. **(A)** Expression of AXL and MER receptors. SC and LC were isolated from 3-week-old wide-type (WT), *Axl* knockout (*Axl* ^−/−^), *Mer* knockout (*Mer* ^−/−^), and *Axl/Mer* double knockout (*Axl* ^−/−^ *Mer* ^−/−^) mice. The expression of AXL and MER was examined by Western blot. **(B)** MuV binding. SC and LC with the indicated genotypes were incubated with 100 MOI of MuV for 1 h on ice. MuV-NP RNA (upper panels) and protein (lower panels) levels were determined by real-time qRT-PCR and Western blot, respectively. **(C)** MuV internalization. SC and LC were incubated with 100 MOI of MuV for 1 h at 37°C. MuV-NP RNA (upper panels) and protein (lower panels) levels were determined. **(D)** MuV replication. SC and LC were infected with 1.0 MOI of MuV. MuV-NP RNA (upper panels) and protein (lower panels) levels were determined at 48 h after infection. **(E)** Intracellular MuV. WT and *Axl* ^−/−^ *Mer* ^−/−^ SC (left panels) and LC (right panels) were infected with MuV as described in **(D)**. Intracellular MuV-NP (red) was localized by IF staining at 48 h. Cellular plasma and nuclei were visualized using IF for α-tubulin (green) and DAPI (blue) counterstaining, respectively. **(F)** MuV loads in culture medium. SC and LC were infected with MuV as described in **(D)**. Virus loads in culture media were measured by TCID_50_ assay at 48 h after infection. **(G)** MuV replication *in vivo*, MuV (1 × 10^7^ PFU) in 10 μl of PBS was injected into each testis of 8-week-old WT and *Axl* ^−/−^ *Mer* ^−/−^ mice (*n* = 3 mice each group). Total RNA was extracted from the testis and relative RNA level of MuV-NP was determined by real-time qRT-PCR. Images represent at least three independent experiments, scale bars, 50 μm. Data are presented as the mean ± SEM of three experiments. ns, not significant; ^*^ *p* < 0.05, ^**^ *p* < 0.01.

Given that AXL and MER inhibit innate antiviral responses in Sertoli and Leydig cells (Sun et al., 2010; Shang et al., 2011), we examined the role of AXL and MER in MuV replication in these cells. Western blot detected AXL and MER in WT Sertoli and Leydig cells (Figure 4A). The AXL and MER proteins were absent in the respective gene knockout cells. Knockout of *Axl* (*Axl* ^−/−^), *Mer* (*Mer* ^−/−^), and both *Axl* and *Mer* (*Axl* ^−/−^ *Mer* ^−/−^), did not affect MuV binding to Sertoli and Leydig cells based on the detection of MuV-NP RNA (Figure 4B, upper panels) and protein (lower panels) levels. Similarly, MuV-NP levels internalized into WT, *Axl* ^−/−^, *Mer* ^−/−^, and *Axl* ^−/−^ *Mer* ^−/−^ Sertoli and Leydig cells were comparable (Figure 4C). However, MuV replication was significantly reduced in *Axl* ^−/−^ *Mer* ^−/−^ Sertoli and Leydig cells 48 h after infection with MuV (Figure 4D). In contrast, a single knockout of either *Axl* or *Mer* did not significantly affect MuV replication. The decreases in MuV replication in *Axl* ^−/−^ *Mer* ^−/−^ cells were confirmed by immunofluorescence staining for MuV-NP (Figure 4E). Accordingly, MuV loads in the culture media of *Axl* ^−/−^ *Mer* ^−/−^ cells were significantly reduced (Figure 4F). These results suggest that AXL and MER are not involved in MuV binding and internalization, but redundantly facilitate MuV replication in Sertoli and Leydig cells. To examine the role of AXL and MER in MuV replication *in vivo*, MuV was locally injected into the testis of WT and *Axl* ^−/−^ *Mer* ^−/−^ mice. Real-time qRT-PCR results show MuV-NP RNA level was significantly increased in WT mice at 48 and 72 h post injection (Figure 4G). In contrast, MuV-NP RNA was insignificantly increased in *Axl* ^−/−^ *Mer* ^−/−^ mice, suggesting that AXL and MER play role in inhibiting MuV replication *in vivo*.

### Inhibition of IFN Expression by AXL and MER

**Figure 5:**
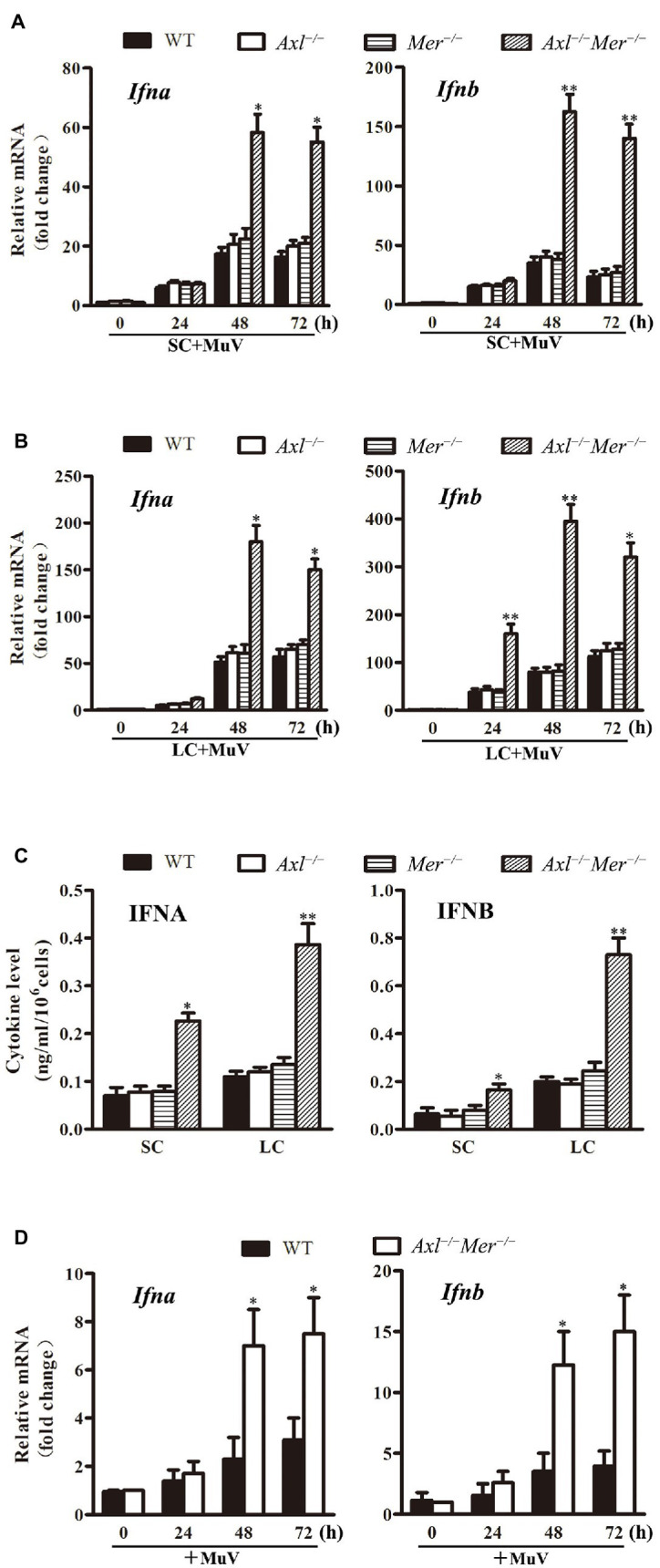
Continued FIGURE 5MuV-induced expression of type 1 interferons (IFN). **(A)** IFN expression in SC. SC were isolated from three-week-old WT, *Axl* ^−/−^, *Mer* ^−/−^ and *Axl* ^−/−^ *Mer* ^−/−^ mice, and were infected with 1.0 MOI of MuV for the specified durations (h). Relative mRNA levels of *Ifna* (left panel) and *Ifnb* (right panel) were determined by real-time qRT-PCR. **(B)** IFN expression in LC. WT, *Axl* ^−/−^, *Mer* ^−/−^ and *Axl* ^−/−^ *Mer* ^−/−^ LC were infected with MuV for the indicated durations. Relative mRNA levels of *Ifna* and *Ifnb* were determined. **(C)** IFN secretion. SC and LC were infected with 1.0 MOI of MuV for 48 h. The secretion of IFNA and IFNB in culture media were determined using ELISA. **(D)** IFN expression *in vivo*. MuV (1 × 10^7^ PFU) in 10 μl of PBS was injected into each testis of 8-week-old WT and *Axl* ^−/−^ *Mer* ^−/−^ mice (*n* = 3 mice each group). Total RNA was extracted from the testis. Relative RNA levels of *Ifna* (left panel) and *Ifnb* (right panel) were determined by real-time qRT-PCR. Data are shown as the mean ± SEM of three experiments. ^*^ *p* < 0.05, ^**^ *p* < 0.01.

Since virus-induced production of type 1 IFNs is the universal mechanism behind the cellular innate antiviral responses that limit virus replication, we examined the expression of IFNA and IFNB in WT, *Axl* ^−/−^, *Mer* ^−/−^, and *Axl* ^−/−^ *Mer* ^−/−^ cells in response to MuV infection. Real-time qRT-PCR results showed that the mRNA levels of *Ifna* and *Ifnb* were significantly higher in *Axl* ^−/−^ *Mer* ^−/−^ Sertoli cells than in WT, *Axl* ^−/−^, and *Mer* ^−/−^ Sertoli cells 48 and 72 h after infection with 1.0 MOI of MuV (Figure 5A). Similarly, MuV induced significantly higher RNA levels of *Ifna* and *Ifnb* in *Axl* ^−/−^ *Mer* ^−/−^ Leydig cells than WT, *Axl* ^−/−^, and *Mer* ^−/−^ cells in a time-dependent manner (Figure 5B). ELISA results confirmed that *Axl* ^−/−^ *Mer* ^−/−^ Sertoli and Leydig cells produced significantly higher levels of IFNA and IFNB in culture media than WT, *Axl* ^−/−^, and *Mer* ^−/−^ counterpart cells 48 h after MuV infection (Figure 5C). Notably, single knockout of either *Axl* or *Mer* did not affect IFN expression, suggesting AXL and MER redundantly inhibit IFN expression in Sertoli and Leydig cells. MuV-induced IFN expression was further examined in the testis after local injection of MuV. The mRNA levels of *Ifna* and *Ifnb* were significantly higher in the testis of *Axl* ^−/−^ *Mer* ^−/−^ mice than WT mice 48 and 72 h post injection (Figure 5D).

### Expression of Antiviral Proteins

**Figure 6:**
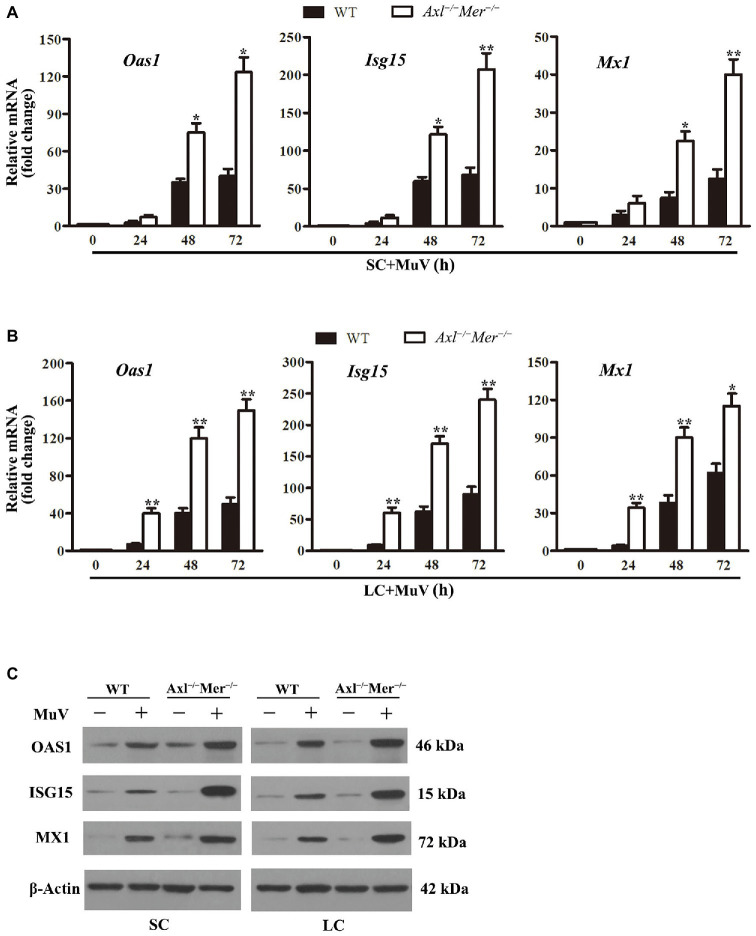
MuV-induced expression of antiviral proteins. **(A)** Expression of antiviral proteins in SC. SC were infected with 1.0 MOI of MuV for the specified durations (h). Relative mRNA levels of 2',5'-ologodenylate synthetase (*Oas1*), IFN-stimulating gene 15 (*Isg15*), and Mx GTPase1 (*Mx1*) were analyzed using real-time qRT-PCR. **(B)** RNA levels of *Oas1*, *Mx1*, and *Isg15* in LC. LC were infected with 1.0 MOI of MuV, and *Oas1*, *Mx1*, and *Isg15* relative mRNA levels were determined using real-time qRT-PCR. **(C)** Protein levels of antiviral proteins. SC and LC were infected with 1.0 MOI of MuV for 48 h. The protein levels of OAS1, ISG15, and MX1 were determined using Western blot. Images of Western blot represent three independent experiments. Data are presented as the mean ± SEM of three experiments. ^*^ *p* < 0.05, ^**^ *p* < 0.01.

Given that IFN-induced antiviral proteins limit virus replication (Sadler and Williams, 2008), we examined the expression of major antiviral proteins, including 2'-5'-oligoadenylate synthetase 1 (OAS1), IFN-stimulated gene 15 (ISG15), and Mx GTPase 1 (MX1), in Sertoli and Leydig cells after MuV infection. While the expression of *Oas1*, *Isg15*, and *Mx1* was dramatically upregulated in both WT and *Axl* ^−/−^ *Mer* ^−/−^ Sertoli cells in a time-dependent manner after MuV infection, their mRNA levels were significantly higher in *Axl* ^−/−^ *Mer* ^−/−^ Sertoli cells than in WT cells at 48 and 72 h (Figure 6A). Similar results were observed in *Axl* ^−/−^ *Mer* ^−/−^ and WT Leydig cells (Figure 6B). Western blot results confirmed that the *Oas1*, *Isg15*, and *Mx1* proteins were remarkably induced by MuV in both *Axl* ^−/−^ *Mer* ^−/−^ and WT Sertoli and Leydig cells at 72 h after MuV infection, and their levels were relatively high in *Axl* ^−/−^ *Mer* ^−/−^ cells compared to WT cells (Figure 6C).

### Role of IFN Signaling in MuV Replication

**Figure 7:**
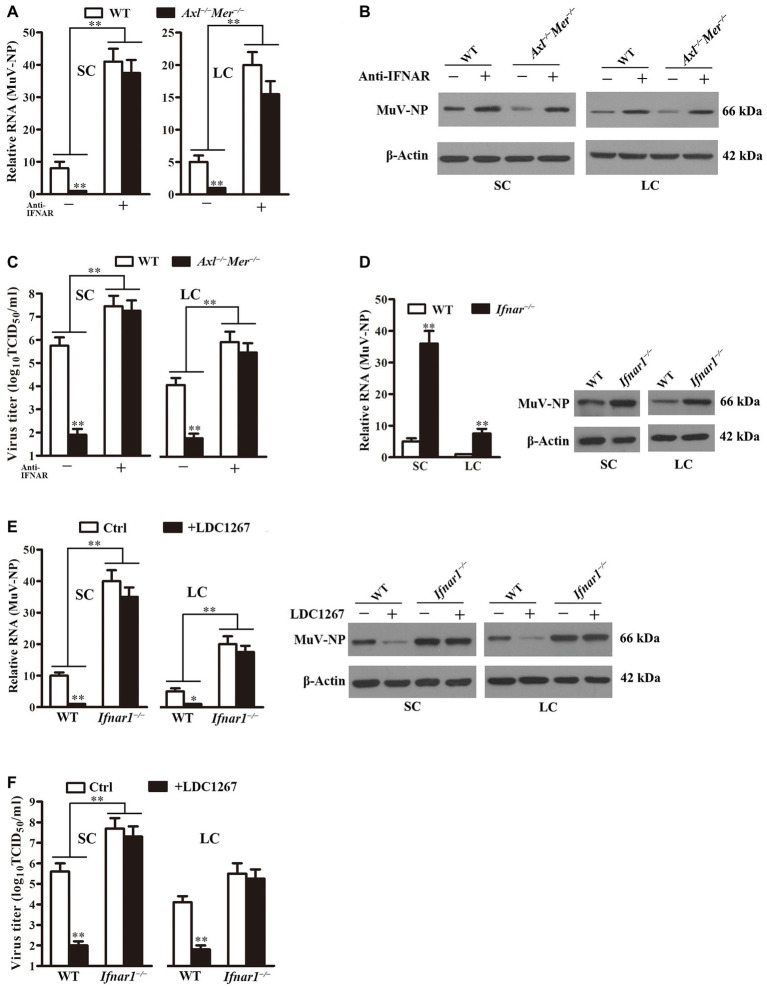
Role of IFN signaling in MuV replication. **(A)** MuV-NP RNA levels. WT and *Axl* ^−/−^ *Mer* ^−/−^ SC, and LC were infected with 1.0 MOI of MuV in the absence (−) and presence (+) of 2 μg/ml neutralizing antibodies against type 1 IFN receptor (anti-IFNAR). MuV-NP RNA levels were determined using real-time qRT-PCR at 48 h after MuV infection. **(B)** MuV-NP protein levels. SC (left panels) and LC (right panels) were treated as described in **(A)**. MuV-NP protein levels were determined using Western blot. **(C)** MuV loads in culture media. SC and LC were treated as described in **(A)**. MuV loads in culture media were measured by TCID_50_ assay. **(D)** MuV replication in type 1 IFN receptor knockout (*Ifnar1* ^−/−^) cells. SC and LC were isolated from three-week-old *Ifnar1* ^−/−^ and WT mice. Cells were infected with 1.0 MOI of MuV. MuV-NP RNA (left panel) and protein (right panels) levels were determined at 48 h post infection. **(E)** Effect of LDC1267, an inhibitor of AXL and MER, on MuV replication. WT and *Ifnar1* ^−/−^ SC and LC were infected with 1.0 MOI of MuV in the presence of LDC1267. The cells were infected with MuV in the absence of LDC1267 served as the controls (Ctrl). MuV-NP RNA (left panels) and protein (right panels) levels were determined using real-time qRT-PCR and Western blot at 48 h post infection. **(F)** MuV loads. SC and LC were treated as described in **(E)**. MuV loads in culture media were determined using TCID_50_ assay. Images of Western blot represent at least three independent experiments. Data are presented as the mean ± SEM of three experiments. ^*^ *p* < 0.05, ^**^ *p* < 0.01.

To determine the involvement of IFN signaling in the MuV replication facilitated by AXL and MER, we assessed the effect of neutralizing antibodies against type 1 IFN receptor (anti-IFNAR) on MuV replication. While MuV-NP RNA was significantly lower in *Axl* ^−/−^ *Mer* ^−/−^ than in WT cells in the absence of anti-IFNAR, the presence of anti-IFNAR increased MuV-NP RNA to comparable levels in both WT and *Axl* ^−/−^ *Mer* ^−/−^ Sertoli (Figure 7A, left panel) and Leydig (right panel) cells 48 h after MuV infection. Western blot results confirmed that the anti-IFNAR remarkably increased MuV-NP protein levels in both WT and *Axl* ^−/−^ *Mer* ^−/−^ cells (Figure 7B). Moreover, the presence of anti-IFNAR significantly increased MuV loads in the culture media of WT and *Axl* ^−/−^ *Mer* ^−/−^ Sertoli (Figure 7C, left panel) and Leydig (right panel) cells 48 h after MuV infection.

The role of type 1 IFN signaling in MuV replication facilitated by AXL and MER was further confirmed using Sertoli and Leydig cells of *Ifnar1* ^−/−^ mice. MuV-NP was significantly increased in *Ifnar1* ^−/−^ Sertoli and Leydig cells at RNA (Figure 7D, left panel) and protein (right panel) levels 48 h after MuV infection. The presence of LDC1267, a common inhibitor of AXL and MER, did not alter MuV replication in *Ifnar1* ^−/−^ Sertoli and Leydig cells while it significantly reduced MuV-NP RNA (Figure 7E, left panels) and protein (right panels) levels in WT cells. Consistently, MuV loads were significantly higher in the culture media of *Ifnar1* ^−/−^ cells than in WT cells (Figure 7F). LDC1267 reduced the MuV loads in the media of WT cells, but did not affect the MuV loads in the media of *Ifnar1* ^−/−^ Sertoli and Leydig cells.

## Discussion

The mammalian testis adopts an immunoprivileged environment for the protection of immunogenic male germ cells from adverse immune responses. The immunoprivileged environment may also provide a sanctuary for microbial pathogens such as viruses to escape immune surveillance (Dejucq and Jegou, 2001; Li et al., 2012). Various virus types have tropism for the testis and impair male fertility (Zhao et al., 2014; Liu et al., 2018). A typical example is MuV, which frequently causes orchitis and may lead to male infertility. The recent outbreak of MuV orchitis is a resurgent threat to male fertility (Philip et al., 2006). Understanding the mechanisms behind MuV infection and replication in the testis can help in the development of preventive and therapeutic strategies for MuV orchitis. In the present study, we elucidated the roles of sialic acid, AXL, and MER in MuV infection and replication in mouse Sertoli and Leydig cells. We found that sialic acid mediated MuV internalization into these testicular cells, whereas AXL and MER facilitate MuV replication. We focused on Sertoli and Leydig cells because these testicular cells can be efficiently infected by MuV and produce pro-inflammatory cytokines and chemokines, which could contribute to the pathogenesis of orchitis (Wu et al., 2016; Jiang et al., 2017).

MuV is a member of the Paramyxoviridae family. Sialic acid plays a role in mediating infection of different paramyxoviruses (Lamb and Parks, 2013). A recent study demonstrated that a trisaccharide with α2,3-linked sialic acid is a receptor mediating MuV infection (Kubota et al., 2016). In the present study, we showed that sialic acid was exposed on the surface of both Sertoli and Leydig cells, and the treatment of these cells with α2,3-Sialidase efficiently removed sialic acid. Coincidently, the sialidase treatment impaired MuV internalization into Sertoli and Leydig cells while MuV binding was not affected by removing sialic acid. These observations suggest that sialic acid plays a role in mediating MuV internalization into Sertoli and Leydig cells, but it is not involved in MuV binding to these cells. The mechanism by which sialic acid facilitates MuV internalization into Sertoli and Leydig cells requires further investigation. In this regard, we should focus on the potential role of sialic acid in promoting the fusion between MuV and target cells through the interplay with viral fusion proteins. Further understanding of the mechanism underlying sialic acid-mediated MuV internalization may be helpful to discover targets for the prevention of MuV infection. It was surprising to find that sialic acid did not affect MuV binding to Sertoli and Leydig cells because sialic acid was supposed to bind MuV as a receptor (Kubota et al., 2016). However, our results are in agreement with recent observations for the role of sialic acid in ZIKV infection, in which sialic acid facilitates ZIKV internalization into target cells but does not affect ZIKV binding (Tan and Huan Hor, 2019). The identification of the specific molecules that mediate MuV binding to testicular cells may also aid in the prevention of MuV infection in the testis.

AXL and MER facilitate infection of a broad spectrum of viruses (Morizono et al., 2011; Meertens et al., 2012; Shibata et al., 2014; Richard et al., 2017). Both AXL and MER are expressed in mouse Sertoli and Leydig cells (Wang et al., 2005). Therefore, we explored the role of AXL and MER in regulating MuV infection in testicular cells. By using *Axl* and *Mer* gene knockout cells, we demonstrated that AXL and MER redundantly facilitated MuV replication in Sertoli and Leydig cells. However, AXL and MER did not affect MuV binding and internalization into the testicular cells. These observations are also helpful in explaining the recent debate regarding to the role of AXL in ZIKV infection. Several studies showed that AXL facilitated ZIKV infection (Fleming, 2016; Meertens et al., 2017; Richard et al., 2017). On the contrary, other studies failed to detect significant differences in ZIKV infection between WT and *Axl* ^−/−^ mice (Wells et al., 2016; Hastings et al., 2017; Wang et al., 2017). The debate that these previous studies raised could have been caused by the use of different models. The studies used *in vitro* cells that express only *Axl* support *Axl*-dependent ZIKV infection (Fleming, 2016; Meertens et al., 2017; Richard et al., 2017). In contrast, the studies using an *in vivo* model show that *Axl* is not indispensable for ZIKV infection (Wells et al., 2016; Hastings et al., 2017; Wang et al., 2017). The discrepancy can be explained by the fact that AXL and MER function in a redundant manner. Our present study confirmed that single knockout of either *Axl* or *Mer* insignificantly affected MuV replication, whereas double knockout of both *Axl* and *Mer* remarkably decreased MuV replication. Therefore, AXL and MER could redundantly facilitate viral replication by dampening the cellular innate antiviral response, rather than mediating viral infection by serving as receptors.

This speculation is also supported by our previous studies showing that AXL and MER redundantly inhibit innate antiviral responses in mouse Sertoli and Leydig cells (Sun et al., 2010; Shang et al., 2011). Given that the expression of type 1 IFNs and subsequently IFN-inducible antiviral proteins in host cells upon viral infection is a key mechanism underlying the restriction of virus replication (Sadler and Williams, 2008; Diamond and Farzan, 2013), we examined the role of IFN signaling in limiting MuV replication in Sertoli and Leydig cells. We demonstrated that the double knockout of *Axl* and *Mer* significantly increased the expression of IFNA and IFNB, as well as major antiviral proteins, including *Isg15*, *Oas1*, and *Mx1*, in Sertoli and Leydig cells in response to MuV infection. *Isg15*, *Oas1*, and *Mx1* limit viral replication by amplifying antiviral signaling, degrading viral RNA, and inhibiting viral gene transcription, respectively (Schoggins and Rice, 2011). Accordingly, disabled IFN signaling using a neutralizing antibody against IFNAR significantly increased MuV replication in both *Axl* ^−/−^ *Mer* ^−/−^, and WT cells. Moreover, MuV replication was significantly increased in *Ifnar1* ^−/−^ Sertoli and Leydig cells. Notably, LDC1267, an inhibitor of AXL and MER did not reduce MuV replication in *Ifnar1* ^−/−^ cells, while it significantly decreased MuV replication in WT Sertoli and Leydig cells. These results suggest that AXL and MER facilitate MuV replication in Sertoli and Leydig cells by inhibiting the antiviral IFN signaling.

The roles of AXL and MER in MuV replication and type 1 IFN induction would not be a specificity of MuV within the paramyxoviridae family and the attenuated strains of MuV are based on following observations: (1) AXL and MER redundantly inhibit IFN expression induced by synthetic RNA analog poly (I:C) that can be produced by various viruses in Sertoli and Leydig cells (Sun et al., 2010; Shang et al., 2011), (2) AXL and MER indeed inhibit the infection of various viruses among different virus families (Morizono et al., 2011; Meertens et al., 2012; Shibata et al., 2014; Richard et al., 2017), (3) AXL and MER could not function as a specific receptor of MuV because they do not affect MuV binding and entry. These observations suggest that AXL and MER facilitate replication of different viruses by inhibiting type 1 IFN production.

In summary, the present study elucidated the mechanisms behind MuV infection and replication in mouse Sertoli and Leydig cells. An α2,3-linked sialic acid mediates MuV internalization into Sertoli and Leydig cells, whereas AXL and MER redundantly facilitate MuV replication within these testicular cells through the inhibition of IFN signaling. These results should aid in the development of preventive and therapeutic approaches for MuV infection in the testis.

## Data Availability Statement

All datasets presented in this study are included in the article/supplementary material.

## Ethics Statement

The animal study was reviewed and approved by Institutional Animal Care and Use Committee of the Institute of Basic Medical Sciences, Chinese Academy of Medical Sciences (Beijing, China).

## Author Contributions

FW and DH designed the experiments. FW, RC, QJ, HW, MG, WL, XY, WZ, and AL performed the experiments. FW, RC, QJ, RH, YC, and DH analyzed data. DH and FW wrote the paper with the other authors providing editorial comments. All authors contributed to the article and approved the submitted version.

## Conflict of Interest

The authors declare that the research was conducted in the absence of any commercial or financial relationships that could be construed as a potential conflict of interest.
